# Development of an NDIR CO_2_ Sensor-Based System for Assessing Soil Toxicity Using Substrate-Induced Respiration

**DOI:** 10.3390/s150304734

**Published:** 2015-02-26

**Authors:** Jasmeen Kaur, Viacheslav I. Adamchuk, Joann K. Whalen, Ashraf A. Ismail

**Affiliations:** 1 Department of Bioresource Engineering, McGill University, 21111 Lakeshore Road, Ste-Anne-de-Bellevue, QC H9X 3V9, Canada; E-Mail: jasmeen.kaur@mail.mcgill.ca; 2 Department of Natural Resource Sciences, McGill University, 21111 Lakeshore Road, Ste-Anne-de-Bellevue, QC H9X 3V9, Canada; E-Mail: joann.whalen@mcgill.ca; 3 Department of Food Science and Agricultural Chemistry, McGill University, 21111 Lakeshore Road, Ste-Anne-de-Bellevue, QC H9X 3V9, Canada; E-Mail: ashraf.ismail@mail.mcgill.ca

**Keywords:** CO_2_ sensor, soil toxicity, substrate-induced respiration, non-dispersive infrared, petroleum

## Abstract

The eco-toxicological indicators used to evaluate soil quality complement the physico-chemical criteria employed in contaminated site remediation, but their cost, time, sophisticated analytical methods and *in-situ* inapplicability pose a major challenge to rapidly detect and map the extent of soil contamination. This paper describes a sensor-based approach for measuring potential (substrate-induced) microbial respiration in diesel-contaminated and non-contaminated soil and hence, indirectly evaluates their microbial activity. A simple CO_2_ sensing system was developed using an inexpensive non-dispersive infrared (NDIR) CO_2_ sensor and was successfully deployed to differentiate the control and diesel-contaminated soils in terms of CO_2_ emission after glucose addition. Also, the sensor system distinguished glucose-induced CO_2_ emission from sterile and control soil samples (*p* ≤ 0.0001). Significant effects of diesel contamination (*p* ≤ 0.0001) and soil type (*p* ≤ 0.0001) on glucose-induced CO_2_ emission were also found. The developed sensing system can provide *in-situ* evaluation of soil microbial activity, an indicator of soil quality. The system can be a promising tool for the initial screening of contaminated environmental sites to create high spatial density maps at a relatively low cost.

## 1. Introduction

Soil pollution due to anthropogenic petroleum hydrocarbon (PHC) spills has become a major environmental hazard. Soil may become contaminated with these hydrocarbons due to their accidental discharge during transportation, leakage from storage tanks and pipeline ruptures [1]. Crude oil spillage has a significant effect on soil properties (e.g., soil pH, hydraulic conductivity, total nitrogen (N), available phosphorus (P)) that reduce the fertility of agricultural soils [2]. In addition, crude oil contaminated soils are proven to be toxic to both flora and fauna [3] and dangerous to human health [4]. Diesel oil is a complex petroleum hydrocarbon derived from crude oil distillation and is made up of low molecular weight alkanes and polycyclic aromatic hydrocarbons (PAHs) [5]. PAHs constitute 5%–30% of diesel oil [6]. In case of an uncontrolled industrial leakage of diesel oil in soil, the fate of PAHs poses a huge impact as they are toxic, carcinogenic and/or mutagenic priority micro-pollutants listed by the U.S. Environmental Protection Agency (U.S. EPA) [7]. Development of technologies to rapidly assess the scope and level of PHC pollution at a relatively low cost and within a short time provides a means of improving management decisions for site remediation.

Common approaches to determine the total concentration of hydrocarbon pollutants in soil focus on sophisticated, time consuming, laboratory-based gas chromatography techniques such as GC/FID, GC/MS and GC/FTIR. However, these methods involve lengthy extraction processes for target contaminants, large volumes of extraction solvents and require instruments with high infrastructure and operating costs [8]. Moreover, the toxic effect of a pollutant in the soil depends not only on its chemical properties and the quantity present, but also upon the amount that is bioavailable, to be absorbed or uptaken by biota [9]. Bioavailability of petroleum hydrocarbons is markedly affected by the soil properties, e.g., soil organic matter content (SOM) and the clay content [10]. Hence, the physico-chemical properties cannot replace biological assessments, which provide insight into the toxicity, synergistic or antagonistic effects of given pollutants [11]. Consequently, a battery of tests on soil biota [12] are used to determine the possible hazards of pollutants on soil ecology and are termed as eco-toxicological tests.

In eco-toxicology testing, the two most frequently used acute toxicity bioassays are the luminescence-based *Vibrio fischeri* test and the immobilization-based *Daphnia magna* test [13]. The extraction of the pollutant into soil leachate is an important step in these tests. Other standard methods include eco-toxicology tests for plants and earthworms developed to measure acute and chronic soil toxicity [14]. Although these tests are useful to identify the bioavailability of pollutants and involve a simple methodology and moderate sensitivity, they require considerable time, cost and expertise and have limited *in-situ* applicability. Hence, the focus of this study was to overcome the limitations of existing methods for soil eco-toxicity assessments.

Petroleum hydrocarbons have a significant impact on microbial community abundance [15], composition and diversity in soil depending upon the degree of hydrocarbon contamination [16]. Bacteria and fungi are susceptible to the toxic effects of such contaminants and once affected disturb the proper functioning of the soil [17]. Thus, the soil microbial activity in a hydrocarbon-contaminated soil can be used as an indicator of the level of the hydrocarbon contamination present in the soil. Assessment of soil respiration by measuring the soil CO_2_ production or O_2_ consumption allows the metabolic activity of soil micro-organisms to be quantified. [18]. Basal respiration (BR) is defined as respiration without adding any organic substrate to soil. The substrate-induced respiration (SIR) involves the measurement of microbial respiration of soil after adding an excess of a readily available nutrient source, usually glucose, to trigger microbial activity [19]. Anderson and Domsch [19] suggested that the respiration rate induced by glucose is proportional to the size of the original soil microbial biomass and hence, can be used as an indicator to determine the microbial biomass in the sample.

The quotient of the actual (basal) and potential (substrate-induced) respiration rate was correlated with PAH concentration at a contaminated site [20]. Margesin *et al.* [21] used SIR as one of the monitoring parameters during the decontamination of a mineral-oil-contaminated soil. Currently, CO_2_ evolved by microbial respiration from soil samples is determined by a simple colorimetric reaction in gas absorbent alkali [22]. This method is cost ineffective when a large number of samples are tested as it involves replacement of the CO_2_ probe with each sample [23].

The reason that SIR could be adapted to a rapid, sensor-based detection method is due to the short time-frame of microbial response. Upon the addition of the substrate, respiration rapidly increases to a maximum and remains at a constant rate for more than 4 h [24]. Anderson and Domsch [19] made CO_2_ emission measurements after 1 h incubation with glucose and correlated them with absolute soil microbial biomass. Lin and Brookes [25] chose 0.5–2.5 h after the glucose addition as the best estimator of the SIR rate. Ananyeva *et al.* [26] recorded CO_2_ emissions within 2–5 h after the application of glucose to evaluate respiration differences between unamended and amended soil samples with added solid and aqueous glucose. Dilly [27] measured CO_2_ emission within 4–24 h after the addition of glucose and targeted the calculation of microbial respiration quotients. The recent studies support a time span of 8–24 h [23,28] to estimate soil carbon (C), N and P mineralization and to correlate soil biological activity with potentially mineralizable N. The other consideration is the soil water content (SWC), which affects substrate diffusion throughout the soil sample and therefore, the reliability of the SIR measurement [29]. Adding a substrate solution instead of a powder gives the best distribution of substrate in soil and is analytically convenient. However, using a substrate solution might cause underestimation of CO_2_ due to its retention in solution [30]. The quantity of substrate needed to achieve a saturated respiration response also needs to be determined for each soil and depends upon the physical and chemical properties of the soil [19].

To improve the efficiency of soil microbial respiration-based methods, a number of simplified CO_2_ sensors are available now [31]. Based on the sensing mechanism, these CO_2_ sensors can be broadly classified as electrochemical (or solid state) and optical sensors [32]. Electrochemical CO_2_ sensors are based on a variety of principles (amperi-, conducto-, and potentiometry) and materials (metal oxides, polymers, ceramics, or sol-gel) [33,34]. They can be further divided into metal oxide [35], NASICON [36] and polymer-based CO_2_ sensors [37]. They make use of micro-electro-mechanical systems (MEMS) and nanotechnologies and are highly sensitive, but suffer from problems of limited measurement accuracy and short-time stability [32]. The most common commercially available CO_2_ sensors are non-dispersive infrared (NDIR) detectors because of their low cost, compact size, easy process control, mass production, and continuous measurement [38].

**Figure 1:**
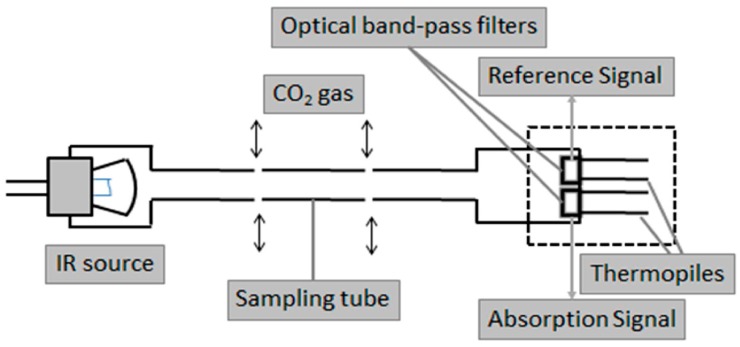
Internal structure of an NDIR CO_2_ sensor, adapted from [40].

NDIR CO_2_ sensors (Figure 1) consist of a pulse-driven IR lamp (*i.e.*, light source), a perforated sampling tube (or chamber), two optical filters, and two IR detectors (thermopiles) [39]. A reflection mirror is attached behind the IR lamp, and an inner wall of the pipe is plated to make emitted IR reach the thermopiles. One thermopile monitors the intensity of light through an optical bandpass filter with 4.0 µm center wavelength, and the other measures the IR absorption due to CO_2_ concentration through an optical bandpass filter with a 4.26 µm center wavelength. The difference of these two raw signals provides the sensor output [40].

The basic principle of NDIR CO_2_ sensors is the energy absorption characteristics of CO_2_ in the infrared region [41]. CO_2_ is known to absorb infrared radiation at wavelengths of 2.7, 4.3 and 15 µm [42]. Generally, the wavelength of the near 4.3 µm region is preferred because of maximum absorption and insignificant interference at this band. The radiation emitted at this wavelength is associated with the carbon dioxide by the Lambert-Beer law [43,44] given by:

(1) $$\frac{I_{d}}{I_{0}}=e^{-\alpha cl}$$

where *I*_d_ is the intensity of the radiation detected at a wavelength of 4.26 µm, *I*_0_ is the intensity if the incident radiation, α is the absorption coefficient of the CO_2_, *c* is the CO_2_ concentration and *l* is the optical path length from the source to the detector.

The availability of these low cost NDIR CO_2_ sensors can be harnessed to measure soil respiration by integrating these sensors into different chamber techniques developed to measure soil CO_2_ efflux *in-situ* due to their small size, low cost, easy maintenance and suitable measurement range. The objectives of this study were (1) to develop and evaluate an NDIR-based CO_2_ sensor system suitable for *in-situ* deployment to measure soil CO_2_ emission in response to the added glucose, based on the SIR method; and (2) to investigate its suitability for toxicity assessment of diesel-contaminated soils.

## 2. Experimental Section

### 2.1. Sensor System Development

The CO_2_ Engine^®^ K30 CO_2_ Sensor (SenseAir, Delsbo, Gävleborg, Sweden) (*Disclaimer*: mention of a trade name, proprietary product, or company name is for presentation clarity and does not imply endorsement by the authors or McGill University, nor does it imply exclusion of other products that may also be suitable) was used for this study. It is inexpensive (under $100 USD) and has a measurement range from 0 to 5000 ppm with an accuracy of ±30 ppm and ±3% of measured value. Its small size (51 × 57 × 14 mm) enabled integration into a small, closed CO_2_ system to be used for the study. Its sampling chamber is a gold-plated labyrinth. No calibration is required during testing because of the built-in self-correcting automatic baseline correction (ABC) algorithm. Infrared CO_2_ sensors are prone to drift of the zero baseline of the calibration curve, which is set by default at the fresh air value of 400 ppm CO_2_. The ABC algorithm is a “low pass filter” that takes advantage of the fact that the CO_2_ level nearly falls to outside fresh air in buildings when unoccupied. It constantly keeps track of the sensor’s lowest reading over a 7.5 day interval (by default) and slowly rescales the sensor probe for any long-term drift detected as compared to the expected 400 ppm CO_2_, hence, updating the sensor calibration regularly.

**Figure 2:**
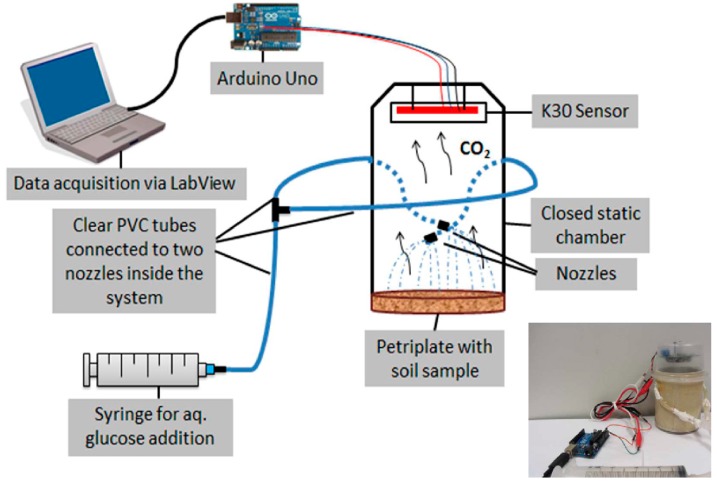
Glucose-induced CO_2_ emission sensing system for soils integrated with NDIR-based K30 CO_2_ sensor.

A small closed static system (non-steady-state non-thorough-flow system) for CO_2_ gas collection and quantification from soil samples was constructed (Figure 2). The K30 CO_2_ sensor was placed on top of the system. To spray glucose solution over the soil sample uniformly, two nozzles attached to two clear PVC (polyvinyl chloride) tubes were integrated into the system. These pipes were attached to a syringe that aided the introduction of the glucose solution into the pipes that fed the glucose to the nozzles. An external fan was used to “cleanse” the sensor between measurements.

Data acquisition software for the K30 sensor was developed using LabView (National Instruments Corporation, Austin, TX, USA) software. An Arduino UNO (Smart Projects, Strambino, Turin, Italy) was used as a microcontroller to power the sensor and receive analog output and was connected to a laptop computer via a USB serial port. Analog voltage data output received from the sensor at 1 Hz was logged in a tab delimited text file. The collected data were analyzed using the Statistical Analysis System (SAS) 9.4 (SAS Institute Inc., Cary, NC, USA) software suite.

**Figure 3:**
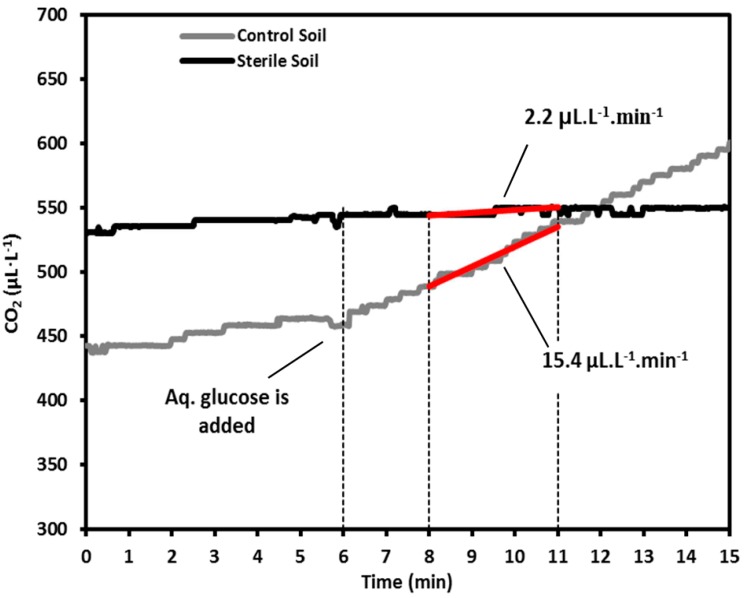
Illustration of the NDIR CO_2_ sensor-based system response for sterile and control samples of organic soil. The glucose solution was added 6 min after placing the system over a petriplate containing soil sample and the CO_2_ concentration data collected between 8 and 11 min (3 min in total) were used to calculate CO_2_ emission.

An illustration of the sensor response for sterile and control samples of organic soil over an extended period of 15 min is shown in Figure 3. For data acquisition, the system was placed over the petriplate containing 20 g of the soil sample, the glucose solution was sprayed 6 min after the start of experiment and the CO_2_ emission data were collected for another 9 min. The data collected between 8 and 11 min (3 min in total), after glucose addition, were used for the calculation of CO_2_ emission∙min^−1^. For regular experiments, glucose was added 1 min after the start of the experiment and hence, the experiment spanned 6 min.

### 2.2. Sensor System Evaluation: Determining the Optimal Glucose Concentration and Soil Moisture Content

**Figure 4:**
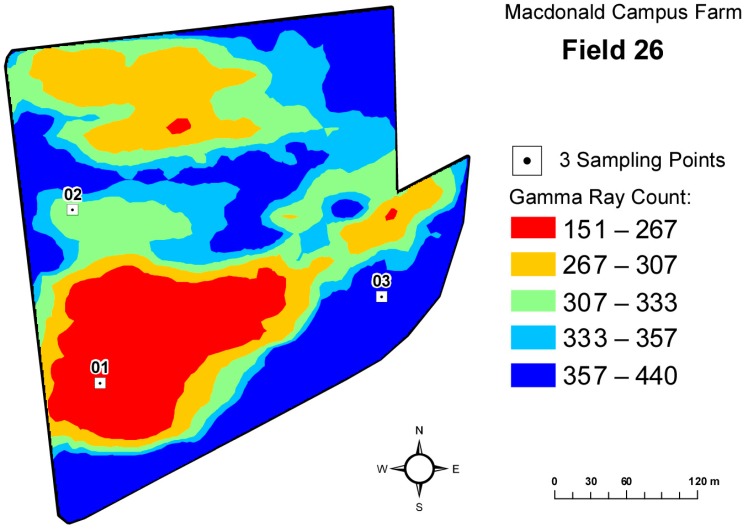
Three sampling locations on the gamma ray count map of Field 26 of the Macdonald Campus Farm, McGill University.

Three soil samples (1: organic, classified as histosol, 2: sandy loam, classified as gleysol and 3: sandy clay loam, classified as gleysol [45]) were collected from Field 26 (Figure 4) of the Macdonald Campus Farm, McGill University, Ste-Anne-de-Bellevue, QC, Canada (45°25′N, 73°56′W) on the basis of variability of the total count derived from gamma-ray spectrometry using SoilOptix^TM^ (Practical Precision Inc., Tavistock, ON, Canada). Key physical and chemical properties of these soil samples are summarized in Table 1. The samples were air dried for 7 days prior to analysis to reduce the contribution of roots to the total SIR response [30], but the moisture content was maintained between 10% and 12% (gravimetric moisture content) to sustain the activity of the soil microbial community. No grinding or sieving of the soils was done, so that the soil aggregate structure was maintained to simulate *in-situ* conditions.

**Table 1:** Physical and chemical properties of collected soil samples.

Sample No.	Sand	Silt	Clay	OM	pH	P	K	Ca	Mg	Al
	g∙kg_mineral soil_^−1^			g∙kg^−1^		mg∙kg^−1^				
1	365	402	233	633	6.9	78	108	10,700	1700	206
2	624	246	130	78	5.9	104	56	1460	190	1082
3	465	280	255	75	7.4	100	124	3600	622	461

Glucose was chosen as a substrate for the study because it can be utilized as a carbon source by most soil microorganisms [46] and was purchased from Sigma-Aldrich Canada Co. (Oakville, ON, Canada). For each soil, five replicates of 20 g air-dry (a.d.) soil were amended with a series of glucose concentrations (0, 5, 10, 15, 20 and 25 mg∙g^−1^ soil) in solution (to adjust soil moisture to 80% of water holding capacity (WHC) of each soil) and CO_2_ concentration was recorded for 5 min with the sensor described in Figure 2, to determine the optimal glucose concentration.

Next, an initial experiment was performed to check the applicability of the designed system towards the soils, with and without microbial activity. All three soils were divided into four sub-samples, consisting of untreated soil for optimal glucose solution addition (control-glucose, CG), untreated soil for deionized water addition (control-deionized water, CD), autoclaved soil for glucose solution addition (sterile-glucose, SG) and autoclaved soil for deionized water addition (sterile-deionized water, SD). Sterilization of soil samples was done by autoclaving the samples 3 times at 121 °C and 15 psi for 1 h on alternate days [47].

### 2.3. Sensor System Evaluation: Toxicity Assessment of Diesel-Contaminated Soil

Five diesel treatments (0, 5, 20, 60 and 150 mg∙g^−1^ of soil) were applied to each soil. Soil samples (500 g each) were spread on aluminum trays to a depth of 1 cm. Diesel fuel (density 836 g∙L^−1^ at 15 °C) was applied over the soil surface by spraying uniformly from a spray bottle, so that it completely covered the surface of the soil with a thin layer of diesel. It was then allowed to penetrate the soil for 5 to 10 min, after that it was mixed thoroughly by hand several times [48]. Soil moisture was then adjusted to 10%–12% (gravimetric moisture content) for all the treatments, including control and diesel-contaminated soils were transferred to 1 kg plastic containers. The pots were covered with loosely fitted perforated lids and were incubated at 22 °C for 7 days.

After incubation, triplicate subsamples (20 g of moist soil) were taken from each control and diesel-contaminated soil and transferred onto Petri plates, for a total of 45 Petri plates (3 replicate subsamples × 5 diesel treatments × 3 soil types). For each soil type, an optimal concentration of glucose (described above) was added into the volume of water required to reach the 80% of WHC of each soil and was sprayed uniformly over the soil sample in the Petri plate. The CO_2_ concentration was determined for five minutes with the sensor system shown in Figure 2, to determine toxicity response.

## 3. Results and Discussion

### 3.1. Glucose Optimization

**Figure 5:**
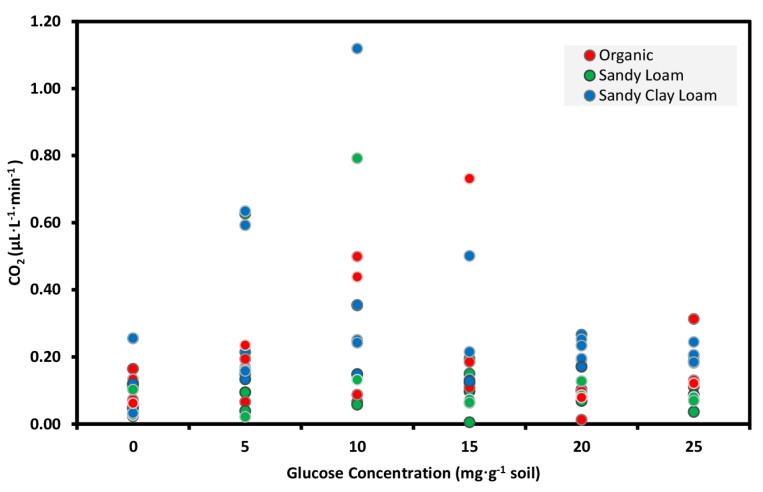
Emission of CO_2_ measured with the NDIR CO_2_ sensor-based system from three soils amended with aq. glucose at concentrations from 0 to 25 mg∙g^−1^ soil. Data represent 20 g of soil samples and five replicates.

Optimizing the glucose concentration for the SIR test with the NDIR CO_2_ sensor-based system showed that the mean score for the 10 mg∙g^−1^ glucose addition was significantly greater (Tukey’s HSD test, *p* < 0.05) than the no sugar control, but no other glucose addition rates were different from the control among the soil types (Figure 5).

The CO_2_ emission rates increased by 2.5-fold, 4.6-fold and 2.8-fold for soil 1, 2 and 3, respectively, when the glucose concentration in solution was increased from 0 to 10 mg∙g^−1^ soil, and decreased with higher glucose concentrations. These findings are consistent with other reports, such as Anderson and Domsch [19], who found that 5 to 50 µM_glucose_∙g_soil solution_^−1^ soil to be optimal for SIR in 12 soils and Ananyeva *et al.* [26] reported 2–15 mg∙g^−1^ induced an optimal respiration response. Further, West and Sparling [30] found optimal respiration rates at 10 mg_glucose_∙g^−1^ soil (60 mg∙mL^−1^ soil water) for three soils and reported a significant decrease in CO_2_ emission after the glucose addition exceeded 10 mg∙mL^−1^ soil water since greater water osmotic potential inhibited respiration. Hence, a glucose concentration of 10 mg∙g^−1^ soil was used to induce SIR for all soils in subsequent tests.

### 3.2. Preliminary Evaluation

**Figure 6:**
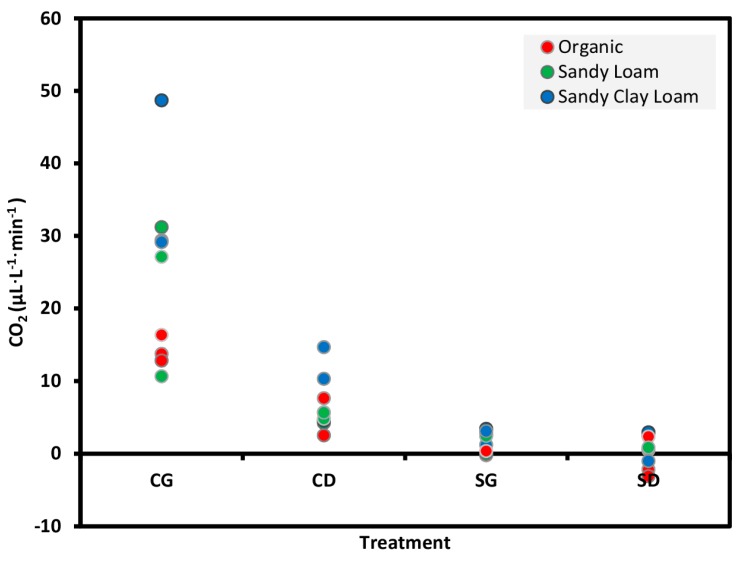
Emission of CO_2_ from three soils measured with the NDIR CO_2_ sensor-based system for the treatments CG: control soil sample added with aq. glucose, CD: control soil sample added with deionized water, SG: sterile soil sample added with aq. Glucose, and SD: sterile soil sample added with deionized water. Data represent 20 g of soil samples and three replicates.

CO_2_ emission measured with the NDIR CO_2_ sensor-based system from the control soils added with aq. glucose (CG) was significantly greater (Tukey’s HSD test, *p* < 0.05) than sterile soil samples added with aq. glucose (SG) for all three soil types (Figure 6; Table 2). Hence, the sensor is able to distinguish CO_2_ emission from soils with variable amounts of microbial activity and the glucose solution incorporation significantly increases the microbial respiration in soils under toxicity stress. The latter proves that the respiration rate of microorganisms increases within a few minutes after adding glucose.

**Table 2:** Analysis of variance of the CO_2_ emission (μL∙L∙min^−^^1^) from sterile and control soil samples.

Source	Degree of Freedom	Sum of Squares	Mean Squares	F value	Pr > F
Treatment (between groups)	11	1.13	0.11	15.16	<0.0001
Error (within groups)	24	0.16	0.01		
Total	35	1.29			

Zero to six hours following the addition of substrate is usually considered as representative of the initial microbial response before biomass growth [49]. Our purpose is to investigate the *in-situ* applicability of an NDIR CO_2_ sensor-based system for evaluation of soil toxicity in terms of diesel amendment. We assumed that the microbes in the toxic soil do not need a long time to metabolize the glucose and their respiration will be triggered quickly. Hence, the CO_2_ emission data between 2 and 5 min after the addition of aq. glucose were used for the measurement of CO_2_ emission from the soil samples.

### 3.3. Diesel Treated Soils Experiment

**Figure 7:**
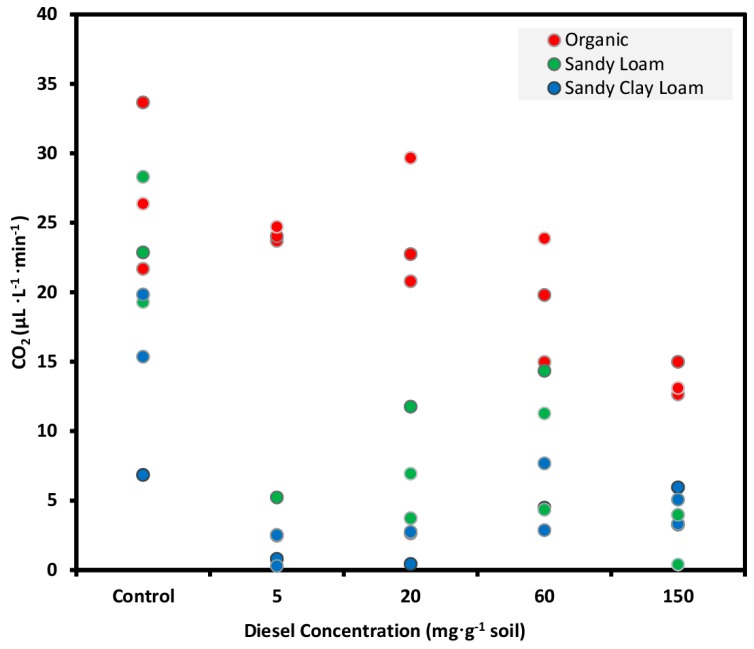
Emission of CO_2_ measured with the NDIR CO_2_ sensor-based system from three soils incubated with water (control) or diesel-contamination for 7 days, prior to the glucose addition. Data represent 20 g of soil samples and three replicates.

The diesel treatment at different rates indicated different CO_2_ emission patterns, in terms of the level of SOM. The mean score of CO_2_ emission measured with the NDIR CO_2_ sensor-based system from control soils was significantly greater (Tukey’s HSD test, *p* < 0.05) from diesel-contaminated soils after incubation period of 7 days (Figure 7; Table 3).

**Table 3:** Analysis of variance of diesel treatment on CO_2_ emission (μL∙L∙min^−1^) from three soil types.

Source	Degree of Freedom	Sum of Squares	Mean Squares	F value	Pr > F
Diesel treatment (between groups)	14	1.03	0.07	19.13	<0.0001
Error (within groups)	30	0.11	0.01		
Total	44	1.13			

Soil 1, organic soil containing 63.3% SOM, showed the microbial SIR rate similar to that of control when diesel contamination was done, showing a slight decrease in respiration with increasing diesel concentration. The presence of high rates of SOM may decrease the accessibility of pollutant to micro-organisms. In such a case, the hydrophobic compounds get partitioned into the organic fraction of the soil and their bioavailability is reduced due to their entrapment in the solid phase of the organic matter [10], hence, decreasing their toxicity towards microbial populations [50].

The soils 2 and 3 (mineral soils) contained 7.8% and 7.5% of organic matter, which implied the soil has a major amount of mineral matter, which acts as an adsorbent [51]. Thus, most of the diesel hydrocarbon was adsorbed on the soil solid; it was bioavailable to micro-organisms and proved detrimental to their survival and activity. Significantly low SIR rates in diesel-contaminated samples of soils 2 and 3 as compared to control samples indicated the inability of the microorganisms to metabolize added petroleum hydrocarbon as a substrate during the first week after the addition. An initial lag phase, during which microbial respiration was inhibited, has also been reported in polluted soil samples with low SOM in previous studies [48,52]. This lag phase suggests that microorganisms need an initial time period to adapt to the presence of hydrocarbons in the medium before using them as substrates; microbial respiration hits a lag phase immediately after the addition of diesel, before its expected increase [16].

The results noted above and the previous literature favor the usage of the SIR and the proposed NDIR CO_2_ sensor-based system for toxicity evaluation of hydrocarbon contaminated soils. Bauer *et al.* [53] found SIR to be inappropriate for the determination of microbial activity of contaminated and non-contaminated soils, but cited low sensitivity of the method used as one of the probable reasons. Many remediation studies have been reported that correlated SIR to the level of organic toxicity present in soil, in addition to other biological indicators. Shi *et al.* [54] found that there were characteristic differences of glucose-induced microbial respirations in the response of contaminated and non-contaminated soils. Pietravalle and Aspray [55] found distinct catabolic diversity between hydrocarbon contaminated soils using multiple SIR assays. Degens and Harris [49] also utilized differences between the SIR responses of microbial communities to simple organic compounds to quantify catabolic diversity of soil microbial communities.

However, the integration of the SIR approach with a NDIR CO_2_ sensor-based system for soil respiration measurements has not been reported yet. Currently, portable infrared gas analyzers (IRGAs) [56] have been integrated in open and closed chambers to develop commercially open (e.g., CFX-1, PP-Systems, Amesbury, MA, USA) and closed chambers (e.g., Li-8100, Li-Cor, Lincoln, NE, USA) to measure soil CO_2_ efflux *in-situ*. The cost of these systems is a major setback as compared to the proposed NDIR CO_2_ sensor-based system.

To put this in further perspective, a longitudinal study on a contaminated site can be done using the sensor system and the correlation of CO_2_ emission data collected with physicochemical parameters can be evaluated over time.

## 4. Conclusions

The application of eco-toxicological indicators to evaluate soil quality is preferable to arbitrary chemical criteria for contaminated soil clean-up initiatives. But the high cost, sophistication and *in-situ* inapplicability of such biological indicators are major setbacks. This paper presented a sensor based approach that indirectly measures potential (induced) biological activity in the soil and hence, could be used to support the physiochemical criteria for site assessment and remediation. The performance of a simple CO_2_ sensing system integrated with an inexpensive NDIR CO_2_ sensor was successfully evaluated to distinguish three different diesel-contaminated and non-contaminated soils. The current design can be modified easily for *in-situ* applicability. The outlook for such research is promising as it eliminates the barriers of *in-situ* evaluation of biological activity of environmental sites. Such methods can serve as promising tools for the initial assessment of the level of contamination and for the determination of highly contaminated areas to be the focus of the final physiochemical evaluation.
